# HIV-1 impairs *in vitro* priming of naïve T cells and gives rise to contact-dependent suppressor T cells

**DOI:** 10.1002/eji.201040377

**Published:** 2010-05-10

**Authors:** Karlhans F Che, Rachel L Sabado, Esaki M Shankar, Veronica Tjomsland, Davorka Messmer, Nina Bhardwaj, Jeffrey D Lifson, Marie Larsson

**Affiliations:** 1 Molecular Virology, Department of Clinical and Experimental Medicine, Linköping UniversityLinköping, Sweden; 2 New York University School of MedicineNew York, NY, USA; 3 University of California at San Diego, Moores Cancer CenterLa Jolla, CA, USA; 4 AIDS and Cancer Virus Program, SAIC Frederick Inc., National Cancer Institute at FrederickMaryland, MD, USA

**Keywords:** DC, HIV-1, Immune responses, Suppressor T-cells

## Abstract

Priming of T cells in lymphoid tissues of HIV-infected individuals occurs in the presence of HIV-1. DC in this milieu activate T cells and disseminate HIV-1 to newly activated T cells, the outcome of which may have serious implications in the development of optimal antiviral responses. We investigated the effects of HIV-1 on DC–naïve T-cell interactions using an allogeneic *in vitro* system. Our data demonstrate a dramatic decrease in the primary expansion of naïve T cells when cultured with HIV-1-exposed DC. CD4^+^ and CD8^+^ T cells showed enhanced expression of PD-1 and TRAIL, whereas CTLA-4 expression was observed on CD4^+^ T cells. It is worth noting that T cells primed in the presence of HIV-1 suppressed priming of other naïve T cells in a contact-dependent manner. We identified PD-1, CTLA-4, and TRAIL pathways as responsible for this suppresion, as blocking these negative molecules restored T-cell proliferation to a higher degree. In conclusion, the presence of HIV-1 during DC priming produced cells with inhibitory effects on T-cell activation and proliferation, *i.e.* suppressor T cells, a mechanism that could contribute to the enhancement of HIV-1 pathogenesis.

## Introduction

DC are professional APC with the unique capacity to prime naïve T cells [1]. Immature DC (IDC) provide a first line of defense against infectious agents, pick up pathogens from tissue and mucosal sites, and migrate to lymph nodes where these cells activate specific naïve T cells. During migration, factors required for tissue homing are downregulated and costimulatory molecules, namely MHC class I and II, CD80, CD86, CD40, and CCR7 are upregulated [2]. These events are crucial for efficient antigen presentation, downstream signaling, and T-cell activation [3].

Mucosal exposure during sexual contact is the primary route of transmission of HIV-1 and DC subsets lining the genital or rectal mucosa are among the first immune cells to come in contact with the virus [4]. Viral binding to DC is mediated by interaction with several surface molecules, *e.g.* the CD4 receptor, CCR5 and/or CXCR4, C-type lectins such as DC-SIGN, and the macrophage-mannose receptor [5]. DC-SIGN binds HIV-1 and enhances infection in newly activated T cells [5, 6] *via* formation of infectious synapses at the DC-T-cell contact zone [7]. The ability of DC to capture HIV-1 and migrate to lymph nodes ensures an environment where there is constant viral presence, especially at the site of DC priming and T-cell activation [1].

HIV-1 infection has a profound impact on the immune system, partly because the virus has evolved to exploit the normal immune functions. The majority of infected individuals with high viral loads have both diminished levels and functionally impaired DC and CD4^+^ T cells [8, 9], which reveals that the presence of high viral burden exerts negative and deleterious effects on host immune cells.

The effects HIV-1 exerts on DC phenotypes and immune functions have been described in various *in vitro* experiments [10–14]. Individual HIV-1 proteins, such as nef, vpr, and tat have been shown to mediate negative effects on immune cells. Nef has been associated with decreased surface expression of MHC class I, CD80, and CD86 molecules in infected cells [15, 16]. Furthermore, nef can upregulate TNF-α and Fas ligand (FasL) expression on DC, resulting in cytotoxic DC with impaired ability to activate CD8^+^ T cells [14]. Vpr downregulates the expression of costimulatory molecules on DC [9], whereas tat triggers IFN responsive gene expression in IDC without inducing maturation [11, 12].

Given the opposing effects observed for HIV-1 proteins, the use of whole virions offers certain advantages when studying the effects of HIV-1 on immune functions *in vitro*. To our knowledge, very few studies have addressed the effects exerted by whole virions on DC [11, 12, 17–19]. Experiments revealed that the production of IL-10 in cocultures with productively infected IDC impaired T-cell proliferation [11] and that p24-positive-infected DC failed to produce IL-12 p70 in response to CD40-ligand activation [19]. Further, infection of plasmacytoid DC induced IFN-α secretion and was partially responsible for the induction of TRAIL on T cells. HIV-1-exposed DC induced apoptosis in both uninfected and infected CD4^+^ T cells due to increased sensitivity to the FasL, TRAIL, TNF-α, and TWEAK receptors [17].

Treg modulate memory T-cell functions in HIV-1-infected individuals [20, 21] and their depletion could lead to immune hyperactivation. It is worth noting that chronic immune-hyperactivation associated with impaired T-cell functions is also seen in HIV-1 infection, although the underlying mechanisms remain elusive. Accumulating evidence suggests that CTLA-4 expression correlate positively with HIV disease progression [22], and that PD-1 expression on CD4^+^ T-cells directly correlates with increased viral load [23]. Nonetheless, blockade of CTLA-4 or PD-1 restored and augmented HIV-specific T-cell functions [22]. Taken together, these studies suggest that failure of T cells to effectively control HIV-1 infection could largely be attributed to the elevated expression of inhibitory molecules.

We investigated the effects of HIV-1 on the ability of DC to prime naïve CD4^+^ and CD8^+^ T cells in an *in vitro* allogeneic system and elucidated the mechanisms through which HIV-1 impairs the ability of DC to prime naïve T cells. We used infectious HIV-1 (inf-HIV) and noninf-HIV chemically inactivated with aldrithiol-2 (AT-2 HIV) virions to determine if productive infection or exposure to virions alone was sufficient to affect DC function. We found that exposure to both inf-HIV and AT-2 HIV impaired the ability of DC to prime naïve T-cell responses. Interestingly, the T cells primed by DC in the presence of HIV-1 suppressed subsequent activation of new naïve T cells. We also found that the suppression was dependent on cell-to-cell contact and independent of inhibitory cytokines, *i.e.* IL-10 and TGF-β. HIV-1-exposed DC showed no major alterations in CD40, CD80, or CD86 expression, whereas the primed T cells had increased expression of proteins known to have a negative impact on T-cell activation and proliferation, such as CTLA-4, PD-1, TRAIL, and Foxp3. The upregulation of CTLA-4, PD-1, and TRAIL, and the signaling events occurring through these receptors appeared to contribute substantially to T-cell impairment as their blockade fully restored T-cell proliferation, fitting with the herein described cell-to-cell contact-dependent mechanism. Our study highlights an important aspect of HIV-1 pathogenesis whereby the presence of HIV-1 virions, whether infectious or noninfectious, during the priming of naïve T cells by DC could have a detrimental outcome on the priming event and therefore contributes to T-cell impairment and immune dysfunctions occurring *in vivo* in HIV-1-infected individuals.

## Results

### Phenotypic characterization of DC and T cells

**Figure 1:**
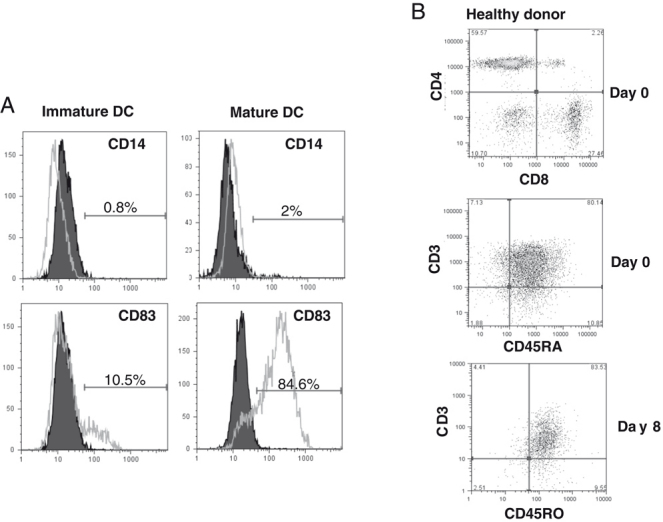
Characterization of IDC and mature DC, naïve, and activated T cells (A) IDC were harvested after 6 days of propagation. In total, 30 μg/mL polyI:C for 24 h was added to induce maturation. DC were characterized by staining with PE-mAb against CD14 and CD83. (A) CD14 and CD83 expression on IDC (left panel) and MDC (right panel)*.* (B) Bulk naïve T cells, negatively selected from PBMC, were immunophenotyped For CD3, CD4, CD8, and CD45RA expression on day 0 for CD4 and CD8 (upper panel), CD3 and CD45RA (middle panel), and after 8 days of MDC–T-cell coculture for CD3 and CD45RO (lower panel).

Several studies have examined the effect of HIV-1-exposed human immature myeloid DC exert on T cells and demonstrated consequential effects, such as production of chemoattractants, proliferation inhibitors, and cytotoxic factors [11–13]. Therefore, we established whether DC exposed to high doses (175–750 ng p24 equivalents) of whole inf-HIV or AT-2-HIV (CXCR4-tropic HIV-MN or CCR5-tropic HIV-1BaL) were impaired in their ability to prime naïve T cells. We established an allogeneic primary culture system consisting of DC and naïve bulk T cells. Inf-HIV-1 and AT-2 HIV-1 were compared to determine whether productive infection was required or if viral exposure alone was sufficient to affect the outcome. The priming consisted of IDC or mature DC (MDC) (Fig. 1A) cocultured with negatively selected naïve CD45RA^+^CD62L^+^ bulk T cells (Fig. 1B). IDC and MDC were CD14^−^ and IDC had no or very low CD83 expression, whereas their mature counterparts had 80% or higher CD83 expression (Fig. 1A). It is worth noting that HIV-1 exposure did not affect DC viability as assessed by Annexin V and Trypan blue exclusion (data not shown). Some studies have shown induction of maturation in Monocyte-derived DC (MDDC) after exposure to high amounts of HIV-1 [24], whereas we and others detected very little or no contribution of HIV-1 to DC maturation [1, 25] (data not shown). The difference in outcome could be due to the virus strains used and/or how the DC were prepared. The T cells displayed a naïve (CD3^+^CD45RA^+^) phenotype and were 90–95% pure. After 7 days of coculture with DC, majority of CD4^+^ and CD8^+^ T cells (80–100%) assumed a memory (CD3^+^CD45RO^+^) phenotype (Fig. 1B).

### Both inf-HIV and non-inf-HIV impaired the ability of DC to prime naïve T cells

**Figure 2:**
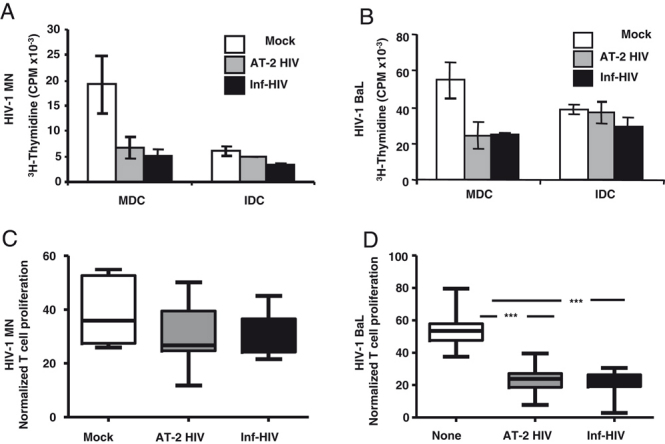
Presence of both inf-HIV and non-inf HIV impairs the ability of DC to prime naïve T-cell responses. IDC and MDC were pulsed with inf-HIV or AT-2 HIV (either HIV-1MN (CXCR4-tropic) or HIV-1BaL (CCR5-tropic)) overnight, washed twice, and cocultured with CFSE-labeled naïve bulk T cells at a ratio of 1:10 (10^4^ DC and 10^5^ T cells). Priming cultures were restimulated with 10 000 DC/well after 7 days of coculture and T-cell proliferation measured on day 8 by CFSE dilution by flow cytometry or by ^3^H-Thymidine incorporation. (A) HIV-1MN effects on the ability of MDC and IDC to induce T-cell proliferation (figure shows one experiment with suppression of T-cell proliferation). Background proliferation with T cells alone ranged from 3 to 8% depending on the donor. (B) HIV-1BAL effects on the ability of MDC and IDC to induce T-cell proliferation (graph shows one representative experiment). (A and B) Bars show the mean of three wells *per* group±SEM. (C and D) Normalized values for assays performed with CXCR4-tropic HIV-1MN (*n*=8) and CCR5-tropic HIV-1BaL (*n*=20) respectively. ^***^p<0.001, one-way ANOVA non-parametric Tukey's test.

High viral loads in HIV-1 infection are implicated in causing DC and T-cell functional impairment [8, 9]. Hence, we investigated the *in vitro* effects HIV-1 has on DC ability to prime T-cell responses. Naïve T cells and IDC or MDC pulsed with mock, AT-2, or inf-HIV-1 (CCR5- or CXCR4-tropic HIV-1; 175–750 ng p24 equivalents/mL: corresponding to ∼0.5–2MOI) were cocultured and T-cell proliferation was assessed at different days by CFSE dilution and/or ^3^H-Thymidine incorporation. Our experiments showed that both CXCR4- and CCR5-tropic AT-2 and inf-HIV-exposed MDC induced less T-cell proliferation as compared with mock DC (CXCR4: Fig 2A and CCR5: Fig 2B). HIV-1-exposed DC inhibited T-cell proliferation by as much as 50% compared with those without HIV-1. Notably, even the lowest HIV-1 dose, *i.e.* 175 ng p24 equivalents/mL, exerted a negative effect on proliferation (data not shown). Nonetheless, the effects at higher doses were more pronounced and consistent in the assays. It is worth noting that these doses do exist *in vivo* where DC–T cell interaction occurs, as 100–10 000 virions (35–3500pg p24) can be released in the contact zone [26].

Although we observed a decreasing trend in T-cell proliferation stimulated by IDC exposed to HIV-1 (Fig. 2A and B), their inherent decreased capacity to activate T cells and ability to suppress T-cell responses [27] masked any significant inhibition caused by HIV-1 exposure. We also found that CXCR4-tropic HIV-1 pulsed DC possessed an impaired ability to induce naïve T-cell proliferation in only 53% of donors/assays tested with an overall insignificance (*p*>0.05) of T-cell suppression when experiments were normalized together (Fig. 2C). Contrarily, the CCR5-tropic HIV-1 had more consistent (95% donors) (*p*<0.001) T-cell impairment after normalization (Fig. 2D). The CXCR4-tropic HIV-1MN showed more diverse effects, which could be due to the difference in pathogenicity attributes between the strains tested. Intriguingly, the CCR5-tropic HIV-1BaL exerted negative effects on mature DC–T cell cocultures independent of the donor, thereby ensures itself and the MDC the prime focus of our subsequent investigation.

### Presence of HIV-1 during priming gave rise to contact- dependent suppressor T cells

**Figure 3:**
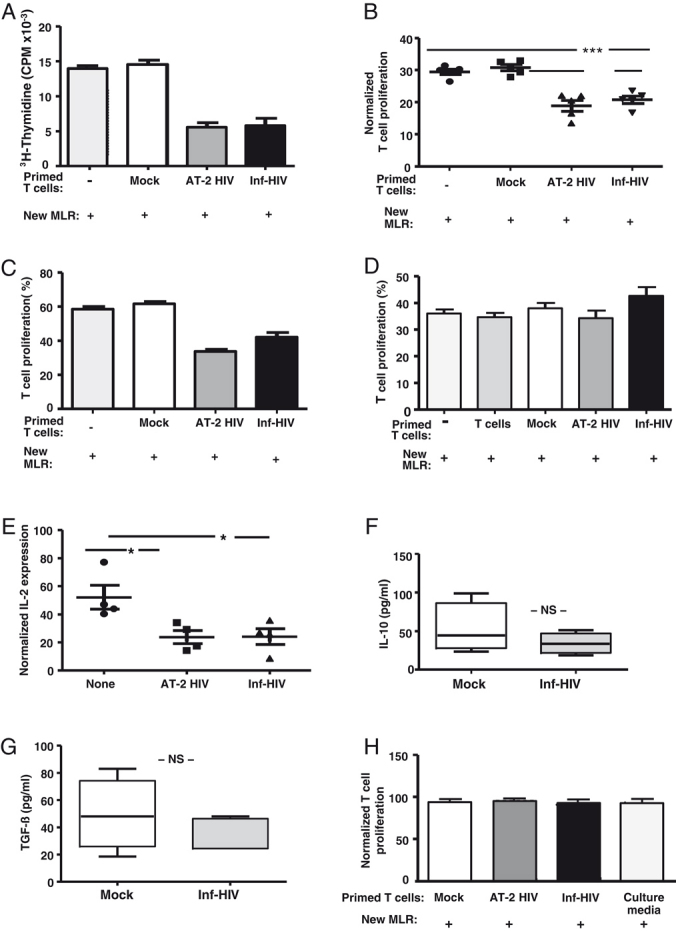
T cells primed in the presence of CCR5-tropic HIV-1BaL impair T-cell proliferation in new naïve priming assays in a contact-dependent manner. (A and B) T cells primed by mock or HIV-1-pulsed MDC were harvested after 8 days, washed, and transferred into new allogeneic cocultures. The ratio of DC to new naïve T cell was 1:10 and 1:1 ratio for the primed and new naïve T cells. T-cell proliferation was measured on day 7 by ^3^H-Thymidine incorporation or CFSE dilution. (A) One representative experiment of T cells primed by mock or HIV-1-pulsed MDC. (B) Normalized values for assays performed (*n*=5). (C) HIV-1-pulsed IDC priming assay (performed as described for (A and B)) with impaired proliferation. (D) Supernatants were harvested from cultures with mock T cells, mock, AT-2 HIV, or inf-HIV-1-pulsed DC and added to new allogeneic cocultures. T-cell proliferation was measured on day 7 by flow cytometry (CSFE) (*n*=5). (E) Supernatants harvested on day 8 from four independent experiments were analyzed by BioPlex IL-2 cytokine assay and results were normalized. (*n*=4). (F–H) Supernatants from day 8 cocultures of naïve T cells pulsed with mock DC, HIV-1-exposed DC, and mock T cells were assessed for IL-10 (F) and TGF-β (G) by ELISA (*n*=6). (H) T cells primed by mock MDC, pulsed with AT-2 HIV, or inf-HIV were harvested after 8 days, washed, and cultured in the upper chamber of a transwell 96-well plate with a new allogeneic DC–T-cell priming culture in the bottom chamber. The ratio of DC to new naïve T cell in the bottom chamber was 1:10, (naïve T-cell numbers equivalent to primed T cells in the upper chamber). T-cell proliferation was measured on day 7 by ^3^H-Thymidine incorporation and flow cytometry (normalized values (*n*=4)). (A, C, and D) Bars show the mean of three wells *per* group±SEM. ^**^ *p*<0.005 and ^***^ *p*<0.001 one-way ANOVA non-parametric Tukey's test.

The diminished proliferation seen for T cells primed by HIV-1-pulsed mature MDDC could be due to countless factors, *e.g.* increased apoptosis or IL-10 production [11]. Therefore, we examined whether memory T cells primed from naïve T-cells or factors secreted in supernatants had any effect on the activation of other naïve T-cells. T cells previously primed by mock, AT-2 HIV, and inf-HIV exposed DC or supernatants derived from these cultures were added to new allogeneic DC–T cell assays and cultured for 7 days. The addition of T cells previously primed by DC in the presence of HIV-1 impaired the proliferation of new naïve T cells (Fig. 3A and B) that was independent of the DC type used (immature or mature) to initiate the previous priming but depended on whether the previously primed T cells exhibited impaired proliferation (Fig. 3C). The maximum levels of T-cell proliferation differed between donors from 45 to 80%. Hence, we showed that the proliferation was significantly impaired in cultures where T cells were previously primed with AT-2 HIV (Fig. 3B: *p*<0.0001) and inf-HIV-exposed DC (Fig. 3B: *p*<0.001).

To determine if soluble factors secreted in the initial priming cultures had an impact on naïve T-cell activation, we added culture supernatants from the initial cultures to new priming cultures. Interestingly, this did not have a negative impact on naïve T-cell priming in the new DC–T cell assays (Fig. 3D). Furthermore, extensive cytokine analysis in culture supernatants failed to divulge any distinct pattern besides decreased IL-2 levels **(**Fig. 3E: *p*<0.05). The decreased IL-2 is suggestive of diminished proliferation seen in T cells primed with HIV-1-pulsed MDC. Given that soluble factors, namely TGF-β, released by Treg type 3 [28], and IL-10, released by type 1 Treg, have the ability to impair proliferation [28], we were particularly interested in determining whether their presence decreased T-cell proliferation in HIV-1-primed cocultures. However, neither TGF-β nor IL-10 levels were significantly affected in the supernatants (Fig. 3F and G). The unaffected levels of TGF-β/IL-10 despite HIV-1 presence ruled out the induction of contact-independent Treg type 3 and Treg type 1 mechanisms. Further, to rule out any possible cytokine degradation or activity loss during the 7-day-long priming cultures, we set up transwell assays whereby HIV-1-primed T cells could constantly supply factors to the new cocultures. Interestingly, we found that the ability to impair new DC–T-cell priming was still curtailed when the cells were separated by a transwell membrane (Fig. 3H). Taken together, our findings point to a cell-to-cell contact-dependent mechanism for the HIV-induced suppression of T-cell proliferation.

### Naturally occurring Treg were not responsible for the HIV-1-pulsed DC-induced T-cell impairment

**Figure 4:**
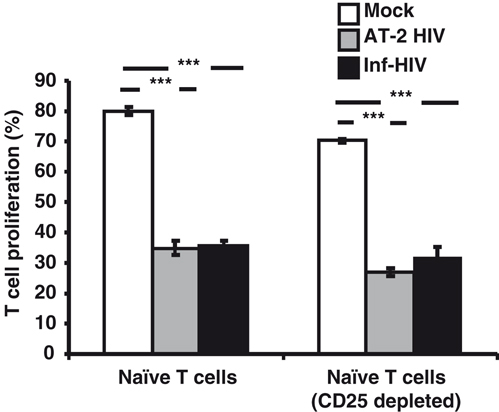
Presence and expansion of naturally occurring CD4^+^CD25^+^ nTreg are not responsible for the impairment of the ability of HIV-1-pulsed DC to prime naïve T cells. MDC pulsed with AT-2 HIV-1 or inf-HIV-1BaL were cocultured with CFSE-labeled naïve bulk T cells or naïve bulk T cells depleted of CD25^+^ cells at a ratio of 1:10. The priming cultures were restimulated with 10 000 DC (same batch of DC used to initiate priming) *per* well after 7 days. T-cell proliferation with or without CD25^+^ cells was measured (graph shows one representative experiment out of four (*n*=4)). Bars show the mean of three wells *per* group±SEM. ^***^p<0.001, one-way ANOVA non-parametric Tukey's test.

Some Treg inhibit T-cell proliferation through secretion of cytokines, *i.e.* TGF-β and/or IL-10, whereas others operate in a contact-dependent manner [29, 30]. Naturally occuring Treg (nTreg) (CD4^+^CD25^+^Foxp3^+^) exist in all individuals [30] and play a critical role in chronic HIV-1 infection [31]. Therefore, we investigated the role of nTreg in the decreased T-cell proliferation seen in HIV-1-exposed cocultures. To address this, we depleted the naïve T cells of nTreg (by removing CD25^+^ T cells), before use. Intriguingly, we found that removal of nTreg failed to restore T-cell proliferation (Fig. 4), indicating their lack of involvement in promoting T-cell inhibition. Our results also suggest that priming of naïve T cells by HIV-1-exposed DC gives rise to contact-dependent suppressor/Treg that are distinct from nTreg.

### T cells primed in the presence of HIV-1 gave rise to increased levels of suppressor T cells

**Figure 5:**
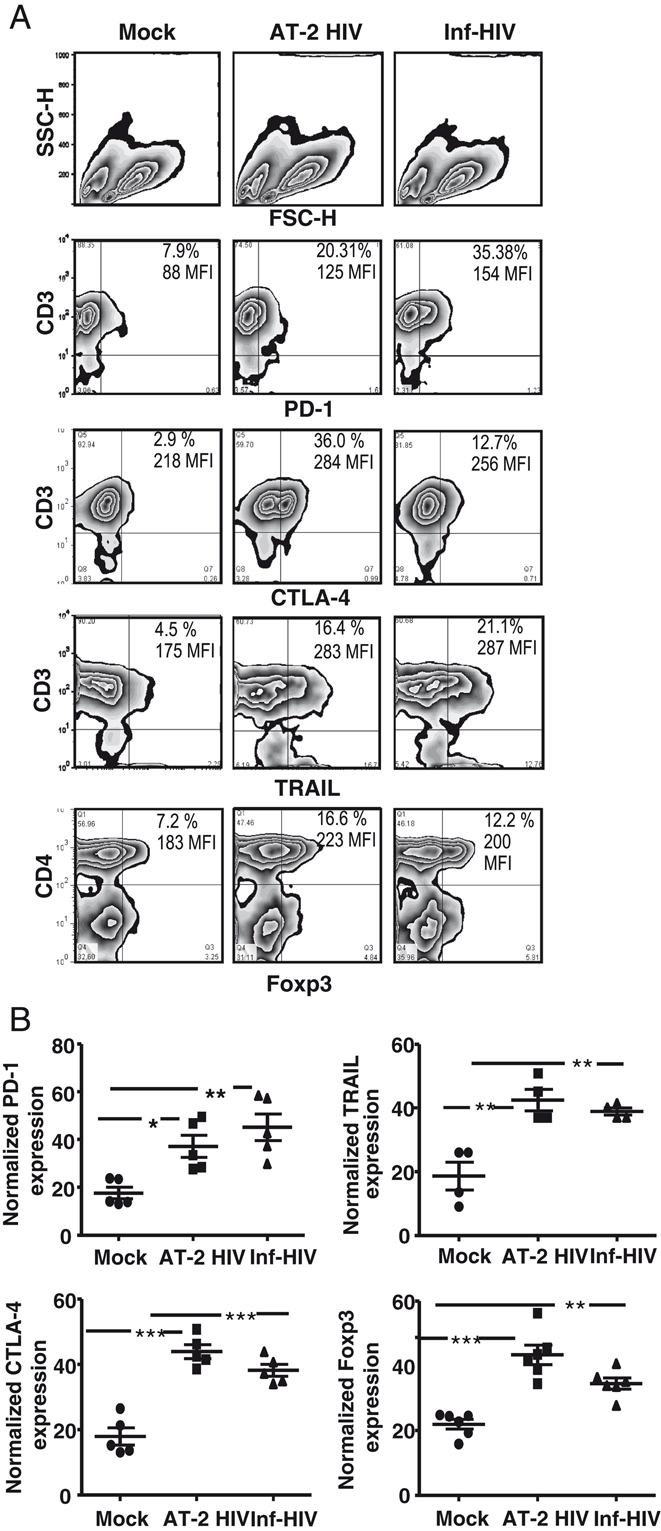
T cells activated and primed by HIV-1-exposed DC upregulate surface expression of certain inhibitory molecules. (A) DC–T-cell cocultures, with or without HIV-1, were harvested after 8 days and immunophenotyped for CD3/PD-1, CD3/TRAIL, CD3/CTLA-4, and CD4/Foxp3. (A) Representative graphs showing percentage expression of PD-1, TRAIL, CTLA-4, or Foxp3 on T cells. (B) Four to six separate experiments were normalized and statistics performed. ^*^p<0.05, ^**^p<0.005, ^***^p<0.001, one-way ANOVA non-parametric Tukey's test.

In HIV-1-infected individuals, several surface markers are involved in dampening T-cell responses, such as CTLA-4, [22] PD-1 [32], and intracellular Foxp3 expression in Treg [31]. Furthermore, TRAIL expression contributed to T-cell impairment in HIV-infected individuals [17]. We determined whether T cells primed in the presence of HIV-1 expressed CTLA-4, PD-1, PD-L1, Foxp3, and TRAIL and found that there was an increase in PD-1 (8–53%), CTLA-4 (8–40%), Foxp3 (5–16%), and TRAIL (7–21%) expressions. The corresponding MFI levels correlated with the percentage of expression (Fig. 5A) on T cells primed with AT-2 HIV and inf-HIV-exposed DC as compared with mock DC. Moreover, both CD4^+^ and CD8^+^ T cells expressed PD-1 and TRAIL, while CTLA-4 was expressed only on CD4^+^ T cells. Foxp3 was found mostly on CD4^+^ T cells, but lower levels occurred on some CD8^+^ T cells (data not shown). It is worth noting that we observed none or very low PD-L1 levels on T cells independent of the culture conditions, whereas both mock and HIV-1-pulsed DC expressed high PD-L1 levels (data not shown). Significant upregulation of PD-1, CTLA-4, TRAIL, and Foxp3 (Fig. 5B) was evident on T cells primed by HIV-1-exposed DC, which emphasizes the critical consequences these molecules have on T-cell proliferation.

### Decreased activation and CD25^+^ levels on T cells primed in the presence of HIV-1-pulsed DC

**Figure 6:**
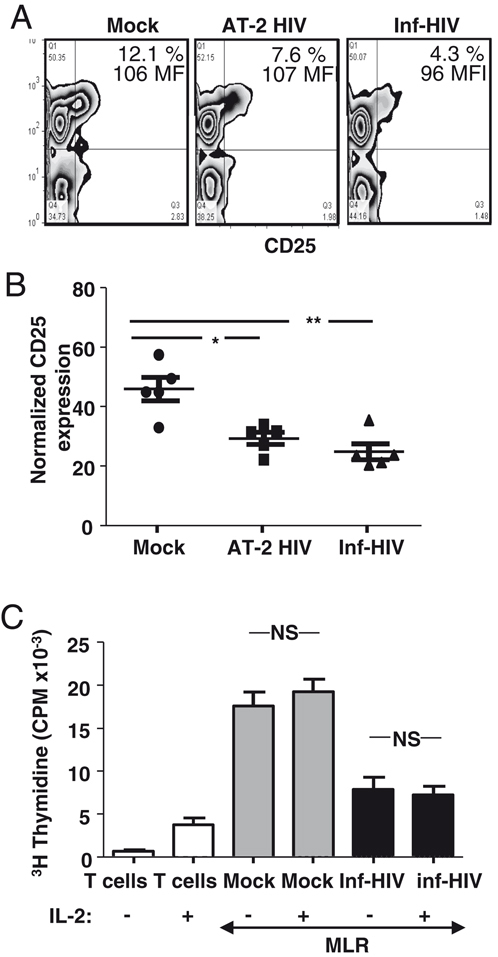
Allogeneic naïve T cells primed in the presence of HIV-1-pulsed DC have decreased frequency of CD25^+^-activated T cells which cannot be reversed by exogenous IL-2. (A) DC–T-cell cocultures with or without HIV-1 were assessed for IL-2Rα (CD25) expression on T cells after 8 days. (B) Five experiments were normalized showing significant differences between mock, AT-2 HIV, and inf-HIV (*n*=5). (C) 150 U/mL IL-2 was supplemented every 2 days for 8 days and proliferation measured (*n*=3). Bars show the mean of three wells *per* group±SEM). ^*^p<0.05, ^**^p<0.005, one-way ANOVA non-parametric Tukey's test.

Activated T cells express CD25 and require IL-2 for survival and proliferation. To address this, we stained the primed T cells for CD25 expression and IL-2 production. Interestingly, there was a significant decrease in CD25 expression (Fig. 6A and B) and IL-2 secretion (Fig. 3E) among T cells primed with HIV-1-exposed DC, which was consistent with decreased T-cell activation observed under these conditions. As we noticed lower IL-2 and CD25 levels among the HIV-1-primed T-cells, we tested whether exogenous IL-2 could rescue T-cell impairment caused by PD-1 or CTLA-4, as shown by others [33, 34]. The addition of IL-2 induced a slight increase in the control T cell and mock DC–T-cell culture proliferations, but had no effect on HIV-1-primed cultures (Fig. 6C, representative experiments, *n*=3), indicating that T-cell impairment in HIV-exposed cocultures could not be salvaged with exogenous IL-2 while the cells were still in contact.

### HIV-1-induced suppression was attributed to surface expression of CTLA-4, PD-1, and TRAIL by T cells

**Figure 7:**
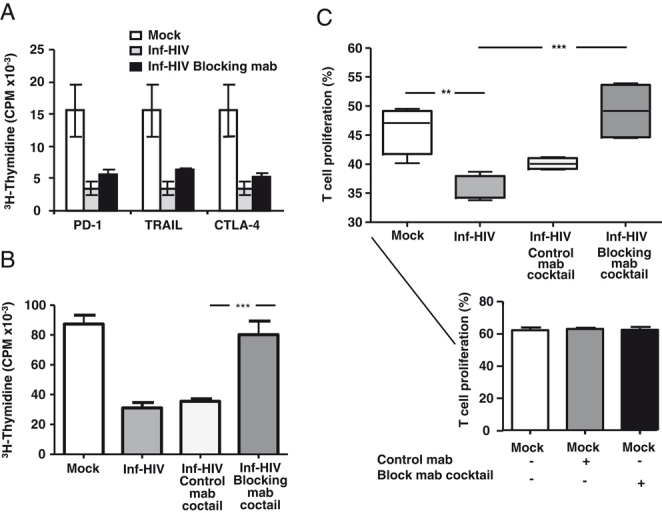
PD-1, CTLA-4, and TRAIL are responsible for HIV-1-induced T-cell impairment. Blocking mAb (20 μg/mL each) PD-1, TRAIL, and CTLA-4 or isotype control were added individually (A), or in combination (B and C) to cocultures of MDC pulsed with inf-HIV-1BaL and CFSE-labeled naïve bulk T cells. The blockers were replenished every third day and T-cell proliferation was measured after 7 days of culture. (B) Graph shows a representative experiment blocking PD-1, TRAIL, and CTLA-4 simultaneously. (C) Four experiments blocking PD-1, TRAIL, and CTLA-4 concurrently were pooled (*n*=4). Effects of PD-1, TRAIL, and CTLA-4 blocking mAb cocktail on T-cell proliferation induced by mock DC (lower panel). (A–C) Bars show the mean of three wells *per* group±SEM. ^**^p<0.005, ^***^p<0.001, one-way ANOVA non-parametric Tukey's test.

The elevated PD-1, CTLA-4, and TRAIL expression by T cells primed by HIV-1-pulsed DC could be the source of T-cell impairment. Therefore, we examined if blocking these molecules effectively salvaged the ongoing HIV-1-induced T-cell suppression [22, 32]. We showed that there was a slight increase, but insignificant restoration of T-cell proliferation when individual blocking mAb were used (Fig. 7A). However, a mAb cocktail against PD-1, CTLA-4, and TRAIL significantly restored T-cell proliferation that ranged from 83% to full recovery (Fig. 7B and C). Thus, HIV-1 presence during DC priming of naïve T cells gives rise to reversible suppressor T cells expressing CTLA-4, PD-1, and TRAIL that could impair the T-cell activation and proliferation.

## Discussion

We demonstrated that HIV-1-exposed DC developed impaired abilities to prime and activate functional T-cell responses from naïve T cells owing to induction of contact-dependent suppressor T cells (expressing PD-1, CTLA-4, and/or TRAIL), which originated from the naïve T-cell pool but not from nTreg. These findings may be relevant to the events preceding HIV-1 pathogenesis *in vivo*, whereby the presence of HIV-1 during the initiation of immune responses against HIV-1 or other pathogens within the lymph nodes may significantly affect the quality and magnitude of the immune responses generated. We previously showed that MDC, when loaded with whole virions, have the ability to prime HIV-1-specific CD4^+^ and CD8^+^ T-cell responses from HIV-1 naïve individuals [25], and expand the existing memory T-cell responses [35]. These studies together with our findings indicate that although HIV-1-pulsed DC could prime and activate antigen-specific T-cell responses, the existence of diverse mechanisms could simultaneously positively or negatively influence ongoing T-cell activation to eventually determine the immune effectiveness generated.

Treg modulate immune activation to prevent pathological self-reactivity [28, 36]. Treg develop either from classical naïve T cells or from nTreg subsets under particular conditions of antigen exposure [28] and mediate suppressive functions *via* direct cellular interaction or secretion of inhibitory cytokines [28]. Importantly, contact-independent regulation could involve participation of immunosuppressive cytokines, *i.e.* TGF-β and IL-10 [36, 37]. It is worth noting that some evidence suggest that IL-10 secretion could result in T-cell impairment induced by HIV-1 [11]. Nevertheless, we ruled out the involvement of IL-10, TGF-β, and other secreted factors occurring in culture supernatants in impairment of T-cell proliferation. Rather, the T cells primed with HIV-1-pulsed DC, inhibited T-cell proliferation in a contact-dependent manner, *via* CTLA-4, PD-1, and TRAIL. Accumulating evidence suggest that reverse signaling in DC after PD-1-PD-L1 ligation triggers T-cell suppression rather than activation [38]. Although we did not detect PD-L1 on T-cells, the possible mechanism could be *via* a cross-talk with PD-L1 expressing DC.

CTLA-4 is upregulated on activated T cells and expressed by Treg to ensure tight regulation of T-cell activation. It antagonizes the signal pathway mediated *via* CD28 ligation leading to T-cell inhibition by reduced IL-2 production [39]. A recent study showed that CTLA-4 and PD-1 were selectively upregulated on HIV-specific CD4^+^ T cells and correlated positively with disease progression [22]. Our study also observed an upregulation and coexpression of both CTLA-4 and PD-1 in HIV-1-primed T-cell cultures. PD-1 is expressed on activated CD4^+^ and CD8^+^ T cells, and B cells, suggesting its involvement in a broader spectrum of immune regulation than CTLA-4, which occurs only on CD4^+^ T cells [32].

Previous studies indicated that PD-1 and CTLA-4 interactions with their respective ligands dampened IL-2 production [33, 34], which was reversed by the blockade of these receptors or exogenous addition of IL-2 [34, 40]. Interestingly, we showed that IL-2 addition failed to overcome the inhibitory mechanisms, whereas blocking of these receptors restored the T-cell proliferation attributes. This could be due to differences between the systems used, such as the sustained maintenance of signaling between DC–T cells and/or the involvement of pathways other than PD-1 and CTLA-4, *e.g.* TRAIL has recently been shown to expand CD4^+^ Treg [41] besides its involvement in T-cell apoptosis [42]. Interestingly, others demonstrated that TRAIL could inhibit antigen-specific T-cell activation without necessarily inducing apoptosis by decreasing calcium influx [43]. TRAIL has two pro-apoptotic death receptors, DR4 and DR5 [15], and three others that could engage TRAIL without initiating apoptosis [44]. Hence, TRAIL expression in the presence of HIV-1 could directly induce the T-cell suppression rather than apoptosis, as shown in our assays.

The mechanisms involved in HIV-1 pathogenesis are complex. Here, we revealed the effects that HIV-1 can have on the ability of DC to prime naïve T cells within lymphoid organs during HIV-1 infection. The HIV-1-induced suppression seen herein seems to depend on the concerted effects of CTLA-4, PD-1, and TRAIL signaling as their concurrent neutralization restored T-cell proliferation. Our finding fits with the fact that CTLA-4 and PD-1 ligation induces immunoinhibitory signals attenuating TCR-mediated IL-2 production and T-cell proliferation [23, 32, 40, 45].

In conclusion, our *in vitro* findings showed that the presence of HIV-1 during the naïve T-cell activation by DC generates suppressor T cells expressing PD-1, CTLA-4, TRAIL, and Foxp3. These molecules were responsible for the T-cell impairment observed, since their blockade successfully restored T-cell proliferation. The induction of contact-dependent suppressor T cells in our system could be either a way for the immune system to control an overload of the system or due to one or several of the immunomodulatory activities of HIV-1 proteins. It is now clear that HIV-1 infection could program DC to generate suppressor T cells. Further studies must be carried out to elucidate the genetic factors and underlying mechanisms that contribute to the expression of inhibitory receptors on T cells after HIV-1exposure.

## Materials and methods

### Culture medium, cytokines, and reagents

RPMI1640 was supplemented with 10 mM HEPES (Fisher Scientific, Leicestershire, UK), 20 μg/mL gentamicin (Fisher Scientific), 2 mM l-glutamine (Sigma-Aldrich, St. Louis, MO), and 1% plasma (DC medium) or 5% heat-inactivated pooled human serum (PHS) (5%) (Fisher Scientific). Recombinant human GM-CSF (Immunex, Seattle, WA, USA) (100 IU/mL) and recombinant human IL-4 (R&D Minneapolis, MN, USA) (300 U/mL).

### DC

Buffy coats from healthy donors were purchased from the transfusion unit at Huddinge Hospital, Stockholm and DC prepared as described previously [18, 25].

DC maturation was induced by adding 30 ng/mL polyI:C (Sigma-Aldrich) and incubated at 37°C in a 5% CO_2_ incubator for 24 h.

### Flow cytometry

PE-mAb directed against CD83, CD86, CD80, CD14, HLA-DR, and their corresponding isotype controls IgG_1_, and IgG_2a_ (BD Pharmingen, Franklin Lakes, NJ, USA) were used for phenotypic characterization of DC. Directly conjugated mAb against PD-1 FITC, PD-1L PE, TRAIL-PE, CTLA-4-PE, CD45RO-PECY5, CD45RA-PECY5, and Foxp3-FITC (BD Pharmingen) were used for T-cell immunophenotyping before and after the coculture. Data acquired on a FACSCalibur (BD Immunocytometry Systems, San Jose, CA, USA) were analyzed using FlowJo software (TreeStar, Ashland, OR, USA).

### Viruses and infection of cells

HIV-1 MN, a CXCR4-tropic clade B virus was produced by infection of CL.4/CEMX174 (T1) cell line. Propagated viruses were purified as described previously [24]. Virus inactivation with AT-2 (AT2 HIV) was performed as described earlier [46]. Inf or AT-2 HIV-1MN or HIV-1BaL (175–750 ng/mL) was added to 1×10^5^ DC and incubated at 37°C for 24 h. Cells were washed twice and used in different assays. Mock DC were used as controls. The DC viability after infection was determined using the Annexin V and 0.4% Trypan blue exclusion.

### Allogeneic DC–T cell proliferation assay

Naïve CD4^+^ and CD8^+^ T cells were negatively selected using magnetic beads by depleting monocytes (CD14), B cells (CD19), NK cell, (CD56), and memory T cells (CD45RO) (Miltenyi Biotec, Auburn, CA, USA) from PBMC. HIV-1-exposed IDC and MDC were harvested, washed twice, and cocultured in 5% PHS with CFSE-labeled (Fisher Scientific) naïve CD4^+^ and CD8^+^ T cells at a ratio of 1:10 (10 000 DC:100 000 T cells) in 96-well plates (BD Falcon, Franklin Lakes, NJ, USA). Assays were restimulated once after 7 days with the same groups of mock, inf-HIV, or AT-2 HIV-exposed DC utilized to initiate the priming event. T cells were analyzed 1 day after restimulation. Mock DC–T-cell cocultures were used as standard to evaluate the effects HIV-1 had on T-cell priming. T-cell proliferation was assessed at several time points between days 3 and 11 by CFSE dilution by flow cytometry and 4 μCi of ^3^H-Thymidine incorporation (Amersham Pharmacia).

### Detection of suppressor/Treg

Day 8 T cells primed by mock, inf-HIV, and AT-2 HIV-exposed DC were added to new mock allogeneic DC–T-cell cultures. DC were cocultured with CFSE-labeled naïve T cells at a ratio of 1:10. The previously primed T cells were added at a ratio of 1:1 to the new naïve CFSE-labeled T cells and proliferation was monitored. Supernatants or T cells from day 8 cocultures primed by mock, inf-HIV, and AT-2 HIV-exposed DC, were added directly to new priming cultures or to the upper chamber, respectively, of a Transwell-96-well system containing new priming cultures at the bottom chamber (pore-size 0.4 μm) (Sigma-Aldrich). Proliferation was measured as described above.

### T-cell proliferation assays with added IL-2

Recombinant human IL-2 (150 IU/mL) (R&D Systems) was added to DC–T-cell cocultures and replenished every second day. The cultures were restimulated after 7 days and T-cell proliferation was monitored on day 8.

### Cytokine measurements

Supernatants from day 8 of cocultures were analyzed by BioPlex Cytokine Luminex assay for IL-10, IL-2, IL-4, IL-5, IL-13, IL-17, TNF-α, G-CSF, TGF-β, IFN-γ, RANTES, PDGF-β, MCP-1, IP-10, bFGF, eotaxia, IL-9, IL-8, IL-6, IL-1R-α, VEGF, and IL-1Rβ (BioRad, Hercules, CA, USA). TGF-β and IL-10 were reexamined individually by ELISA (eBioscience, San Diego, CA, USA).

### Proliferation assays with blockade of PD-1, CTLA-4, and TRAIL

CFSE and ^3^H-Thymidine assays were performed as described above. Briefly, the different DC–T-cell cocultures were incubated with 20 μg/mL isotype control antibody (normal-naïve goat total IgG) or anti-human TRAIL/TNFSF10, IgG_2b_ or anti-human PD-1 antibody (R&D), or 20 μg/mL of isotype control antibody IgG_2ak_ (G155–178) or anti-CTLA-4 antibody (BN13; BD). The cultures were maintained for 7 days in 5% PHS before T-cell proliferation was measured.

### Statistical analysis

We experienced a considerable degree of variability within the experiments and hence statistic values were obtained by normalizing or transforming the data. Each value within a group was divided by the sum total of values within the group and presented as percentage. The GraphPad PRISM software (La Jolla, CA, USA) was used for data analysis using one-way ANOVA nonparametric Tukey's test to compare (^***^p<0.001, ^**^p<0.005, ^*^p<0.05) between the groups.

## Abbreviations

AT-2 HIV: HIV-1 chemically inactivated with aldithiol-2
IDC: immature DC
inf-HIV: infectious HIV-1
MDC: mature DC
MDDC: Monocyte derived DC
nTreg: naturally occuring Treg
PHS: pooled human serum
